# *Epichloë* endophytes improved *Leymus chinensis* tolerance to both neutral and alkali salt stresses

**DOI:** 10.3389/fpls.2022.968774

**Published:** 2022-10-18

**Authors:** Lijia Yin, Maoying Wei, Guanghong Wu, Anzhi Ren

**Affiliations:** ^1^ Tianjin Key Laboratory of Water Resources and Environment, Tianjin Normal University, Tianjin, China; ^2^ College of Life Sciences, Nankai University, Tianjin, China

**Keywords:** endophyte, *Epichloë bromicola*, *Leymus chinensis*, neutral salt stress, alkali salt stress

## Abstract

Symbiotic relationships with microbes may influence how plants respond to environmental change. In the present study, we tested the hypothesis that symbiosis with the endophytes promoted salt tolerance of the native grass. In the field pot experiment we compared the performance of endophyte-infected (E+) and endophyte-uninfected (E−) *Leymus chinensis*, a dominant species native to the Inner Mongolia steppe, under altered neutral and alkaline salt stresses. The results showed that under both neutral and alkaline salt stresses, endophyte infection significantly increased plant height, leaf length and fibrous root biomass. Under neutral salt stress, endophyte infection decreased Na^+^ content and Na^+^/K^+^ ratio (*p*=0.066) in the leaf sheath while increased Ca^2+^ and Mg^2+^ content in the rhizome. Under alkali salt stress, endophyte infection tended to increase K^+^ content in the fibrous root, enhance Mg^2+^ content in the fibrous root while reduce Na^+^/K^+^ ratio in the leaf blade in the 100 mmol/L alkali salt treatment. Although endophyte-infected *L. chinensis* cannot accumulate Na^+^ high enough to be halophytes, the observed growth promotion and stress tolerance give endophyte/plant associations the potential to be a model for endophyte-assisted phytoremediation of saline-alkaline soils.

## Introduction

Soil salinization is a widespread issue throughout the world. It is estimated that more than 6 percent of the world’s land and 30 percent of the world’s irrigated areas suffer from salinity problems (Chaves et al., 2009). Soil salinization generally includes two kinds of stresses: neutral salt stress resulted from NaCl and Na_2_SO_4_, and alkali salt stress resulted from NaHCO_3_ and Na_2_CO_3_. Plants grown in neutral salt-affected soils are mainly subjected to water deficit, ion toxicity, and disorders of mineral nutrients (Reza Sabzalian and Mirlohi, 2010). Excess amount of salt ions in soils can damage plant roots as well as the aboveground plant parts, and appear to be a major constraint to plant and crop productivity (Parihar et al., 2015; Liu et al., 2022). Alkali salt stress causes not only the detrimental salt stress, but also induces high pH stress. The high pH soil surrounding the roots directly reduces the root activity, interferes with ion uptake, and breaks intracellular ion balances in plants (Guo et al., 2009; Kaiwen et al., 2020).

Saline soil rehabilitation can be performed by non-plant based, environment-friendly modifications (e.g., structural engineering alterations, leaching of salts) and plant-based remediation (e.g., phytoremediation) (Gutierrez-Gines et al., 2016). Besides halophytes, the use of plant varieties with elevated tolerance to salinity is another alternative in phytoremediation (Yin et al., 2014). Several studies have demonstrated that the local adaptation of plants to their environment is usually driven by closely associated microbes (Rodriguez and Redman, 2008; Porcel et al., 2011) and it has been documented that mutualistic symbiosis with rhizobia (Franzini et al., 2019) and arbuscular mycorrhizal fungi (Rodriguez et al., 2004; Ma et al., 2021) can reduce the negative effects of salinization and improve the salt tolerance of plants grown in saline soils. Mutualistic microbiota symbiosis may improve water absorption and osmotic regulation of host plants by alleviating the adverse effects of excess salt ion accumulations in host plants (Franzini et al., 2019).

*Epichloë* endophyte is a class of symbiotic fungi and mainly exists in the aboveground part of the plants. Plants serve as hosts and provide nutrients to their endophytes, and endophytic fungus protect them from biotic and abiotic stresses (Xia et al., 2015; Bastias et al., 2017; Shi et al., 2020), especially drought (Ren et al., 2011; Wang et al., 2015; Decunta et al., 2021; Manzur et al., 2022). Endophytic fungus confer drought tolerance to the host grass by increasing root growth, enhancing photosynthesis and osmotic adjustment (Richardson et al., 1992; Malinowski et al., 1998; Decunta et al., 2021). When growing in saline soils, plants are also exposed to drought stress. But limited studies about the effect of endophyte infection on salt tolerance of host grasses have focused on the neutral salt stress in a hydroponic system. In our previous study, we found that endophyte infection could significantly improve the tolerance of tall fescue to NaCl solution, a common lawn grass, by enhancing plant biomass and Na^+^ uptake ability of the host grass (Yin et al., 2014). Recently, Chen et al. (2021) found that endophyte infection ameliorated adverse effects of NaCl solution on *Hordeum brevisubulatum*, an important forage crop, by increasing the conducting tissues and endodermis thickness, which may help inhibiting water loss and the decrease of transport capacity. Endophyte-infected species occur in almost all habitats where grasses are common, including a large number of wild grasses, pasture grasses, lawn grasses, cultivated grains and their wild relatives, as well as weed grasses (Bacon, 1993; White, 1993; Clay and Schardl, 2002). The expression of salt tolerance in a saline hydroponic system could be different from expression in a saline soil-based system (Tavakkoli et al., 2012). Moreover, salinization and alkalization frequently co-occurred in soils, and the alkali stress expressed more serious growth inhibition than the neutral salt stress. However, little is known about whether endophyte infection can also improve the alkali tolerance of host plants.

*Leymus chinensis*, an important perennial rhizome grass, is widely distributed at eastern Eurasian steppe zone, from North Korea westward to Mongolia and northern China, and northwestward to Siberia. Due to excellent stress tolerance, rapid growth, high palatability and herbage production, *L. chinensis* is an economically and ecologically important forage grass (Lin et al., 2017; Liu et al., 2017). In the present study, endophyte-infected (E+) and uninfected (E−) *L. chinensis* were planted under neutral salt ranging from 0 to 400 mmol/L treatments or alkali salt ranging from 0 to 300 mmol/L treatments. The changes in the plant growth and photosynthesis, biomass allocation, and the accumulation of Na^+^, K^+^, Ca^2+^, Mg^2+^ and Na^+^/K^+^ in various plant parts were tested. Specifically, the following questions were addressed: (1) does neutral salt tolerance also exist in endophyte-infected wild grasses in soil-based system? And (2) does the endophyte improve alkali tolerance in the host grasses?

## Materials and methods

### Plant materials and growth conditions

In the middle and northeast parts of the Inner Mongolia steppe, *L. chinensis* is one of the most important dominant species. In the previous survey made by our lab (Wei et al., 2006; Zhu et al., 2013), the endophyte infection rate of *L. chinensis* in the Abaga Banner population was about 63.3%. Endophyte has been identified as *Epichloë bromicola* (Zhu et al., 2013). Thirty E+ and 30 E− plants were originally transplanted from different individuals in the same population from Abaga Banner of Inner Mongolia (43.90° N, 115.34° E). To avoid misleading 30 E+ and 30 E− plants were selected and maintained in the experimental field at Nankai University. After two years’ growth we randomly sampled tillers of approximately equal size for this experiment. During their growth, we clipped the plants repeatedly and kept them in vegetative growth. For this experiment, we transplanted 10 tillers of approximately equal size into a white plastic pot (23-cm diameter and 25-cm depth) filled with 5 kg of fluvial sand. Before conducting experiments, fluvial sand was washed with distilled water until the conductivity of the eluate was close to that of distilled water, and endophyte infection status were confirmed microscopically by examining leaf sheaths of each plant for the presence of fungal hyphae after staining with lactophenol aniline blue (Latch et al., 1984). The pots were all located in the experimental field at Nankai University, with transparent flashing above them. Plants were subjected to ambient light and temperature regimes. The plant growth conditions were maintained at a mean photoperiod of 15/9 h (light/dark) and a mean air temperature of 35/24°C (day/night) with an approximate 65% relative humidity. The positions of the pots were randomly rotated each week to minimize location effects. Salt Treatment

Here two neutral salts and two alkaline salts were considered simultaneously base on the salt components and characteristics of alkaline soil in northern China. A mixture of NaCl and Na_2_SO_4_ (molar ratio, 1: 1) was withheld to induce neutral salt condition, and another mixture of NaCl, Na_2_SO_4_, NaHCO_3_, and Na_2_CO_3_ (molar ratio, 1: 9: 9: 1) was withheld to induce alkaline salt treatment. Neutral salt treatment included four levels: 0, 200, 300, and 400 mmol/L. Alkali salt treatment included four levels: 0, 100, 200, and 300 mmol/L. Four salts concentrations were selected according to the tolerability of *L. chinensis* cultivar to the salt–alkaline stress and the varying ranges of salinity and pH in the soil. Each treatment was replicated five times. Different salt levels were achieved by the addition of distilled water containing the corresponding concentration of mixed salt. The control group was watered with distilled water. The salt solution was added once every two days, and its amount was 3 times the water field capacity of the planting sand so as to wash away the previous accumulated salt. Every morning, a soil moisture probe (ECH_2_O Check, Decagon Devices, Pullman, WA, USA) was inserted to a depth of 5 cm into each pot to record the volumetric water content percentage, and the lost water was supplemented with distilled water to maintain the soil water content at the field water capacity level. Two hundred milliliters Hoagland complete nutrient solution was supplemented twice a week. The experiment was performed from May 10 to August 23, and lasted 105 d.

### Growth parameters and metal ions measurements

The tiller number, leaf number, plant height, leaf length and leaf width of each pot were counted or measured at the beginning and end of the experiment. At the end of the experiment, grass samples were separated into the aboveground plant parts (leaf blade and leaf sheaths) and the belowground plant parts (fibrous roots and rhizomes), weighed after drying in the oven at 80°C. Afterwards, the oven-dried samples were crushed with a grinder, and then placed in a muffle furnace and ashed at 650°C. The samples were digested with 5% nitric acid. The contents of Na^+^, K^+^, Ca^2+^, and Mg^2+^ in the solutions were determined by an inductively coupled plasma - optical emission spectrometry (ICP-OES, Perkin Elmer Optimal 8300).

### Physiological indicators measurements

Specific leaf area (SLA) was calculated using two fully unfolded new leaves for each pot to measure their area and weigh their fresh and dry weights. Leaf moisture content (LWC) was calculated by fresh weight and dry weight of leaves. Net photosynthesis (*P*_n_) was measured on second or third leaves using a portable photosynthesis system (LI-6400, LI-COR, Lincoln, USA). Leaf nitrogen content (LNC) was determined with an elemental analyzer (vario MACRO cube, Elementar, Germany). Photosynthetic nitrogen use efficiency (PNUE) was calculated as the ratio of CO_2_ assimilation rate to leaf nitrogen content. Chlorophyll *a*, chlorophyll *b*, and carotene in fresh leaves were extracted with solution (absolute ethanol and acetone, 1:1), and were assayed in a spectrophotometer (UV1800, Shimadzu, Japan) at wavelengths of 440, 645, and 663 nm. Photosynthetic pigment content was measured according to the procedure of Lin et al. (1999).

### Statistical analyses

Data were analyzed using SPSS 16.0 statistical software (SPSS Inc., Chicago, IL, USA). The differences between endophyte-infected (E+) plants and uninfected (E−) plants, and between neutral salt or alkali salt treatments were tested by one-way ANOVA LSD test at a confidence interval of 95%. Interactions between endophyte status (E) and neutral salt stress (NSS) or alkali stress (ASS) on growth parameters and physiological indexes were tested by two-way analysis of variance (ANOVA) at a confidence interval of 95%.

## Results

### Plant growth and biomass allocation

**Figure 1:**
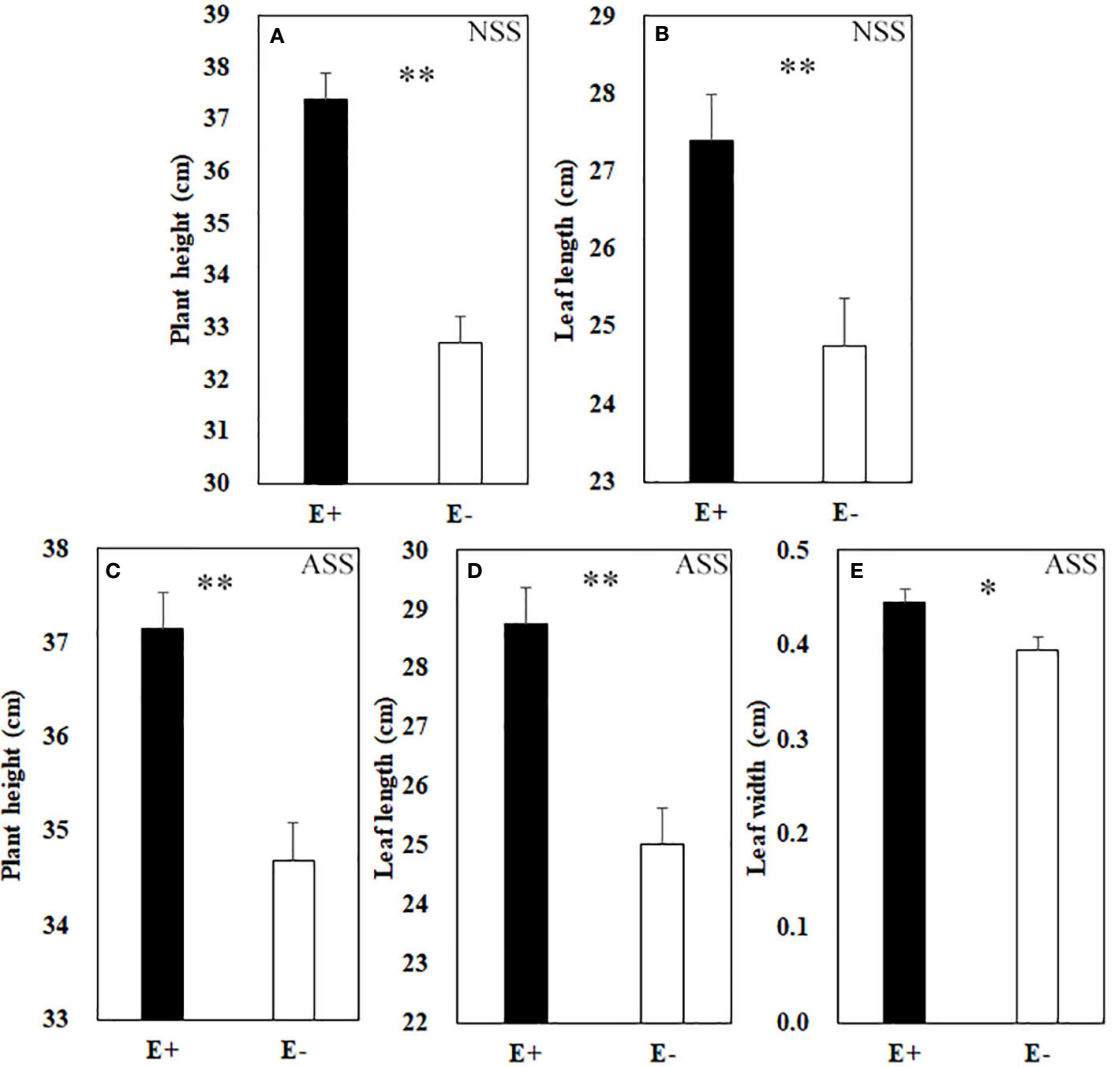
Plant height **(A)** and leaf length **(B)** under neutral salt stress (NSS) as well as plant height **(C)**, leaf length **(D)** and leaf width **(E)** under alkaline salt stress (ASS) of endophyte infected (E+) and uninfected (E−) *L. chinensis*. Values are mean ± standard error. * and ** indicate significant difference at *p* < 0.05 and *p* < 0.01, respectively.

At the end of the experiment, all plants except those treated with 300 mmol/L alkali salt survived, therefore we did not include 300 mmol/L treatment in the result. Both neutral salt and alkali salt stresses significantly inhibited tiller number, leaf number, plant height, SLA and LWC of *L. chinensis*, only alkali salt stress was more serious than neutral salt stress (**Tables 1**, **2**). Under neutral salt treatments, endophyte infection significantly increased plant height and leaf length of the host (**Figures 1A, B**). Under alkali salt treatments, endophyte infection significantly increased plant height, leaf length and leaf width of the host (**Figures 1C–E**). The effect of endophyte status on the vegetative growth of *L. chinensis* in the same concentration (200 mM) of NSS and ASS treatments were shown in **Table 2**. The presence of endophyte significantly increased the plant height of *L. chinensis*, but neutral salt stress decreased the plant heights, and alkali salt stress decreased further. Salt stress decreased leaf length and width, and changed the size or area of leaf.

**Table 1:** ANOVA results for the effect of endophyte status, salt stress on vegetative growth of *L. chinensis*.

		Plant height	Tiller number	Leaf number	Leaf length	Leaf width	Ratio of leaf length to width	SLA	LWC
E	F	41.239	4.215	2.728	9.338	0.811	2.479	3.767	3.010
	*p*	**<0.001**	0.973	0.367	**0.005**	0.374	0.125	0.211	0.803
NSS	F	36.072	1.696	51.231	9.684	5.779	0.585	11.98	23.22
	*p*	**<0.001**	**0.001**	**<0.001**	**<0.001**	**0.003**	0.629	**0.003**	**0.004**
E×NSS	F	0.201	1.636	1.236	0.422	1.130	0.791	1.356	1.794
	*p*	0.895	0.345	0.181	0.739	0.352	0.508	**0.006**	0.212
E	F	19.567	0.937	1.388	18.118	6.215	0.507	9.607	12.77
	*p*	**<0.001**	0.731	0.908	**<0.001**	**0.020**	0.483	0.345	0.414
ASS	F	127.412	3.183	**53.318**	15.328	37.286	7.041	6.703	12.88
	*p*	**<0.001**	**<0.001**	**<0.001**	**<0.001**	**<0.001**	**0.004**	**<0.001**	**<0.001**
E×ASS	F	11.827	1.422	1.583	1.385	0.679	0.352	0.470	0.456
	*p*	**<0.001**	0.259	0.915	0.270	0.516	0.707	**0.013**	0.661

E, endophyte; NSS, neutral salt stress; ASS, alkali salt stress. Significant effects (p ≤ 0.05) are shown in bold.

**Table 2:** The effect of endophyte status on the vegetative growth of *L. chinensis* in the same concentration (200 mM) of NSS and ASS treatments.

Salt concentration	Endophyte	Plant height(cm)	Leaf length (cm)	Leaf width (cm)	Ratio of leaf length to width	SLA(cm^2^/g)	LWC(%)
0 mM	E+	43.8 ± 0.6A	31.1 ± 0.9A	0.57 ± 0.02A	55.7 ± 3.2C	181.1 ± 3.9A	65.5 ± 0.6A
	E−	38.7 ± 0.2B	28.8 ± 0.5AB	0.51 ± 0.02AB	57.3 ± 2.1C	158.5 ± 3.7B	64.9 ± 0.6A
200 mM NSS	E+	36.5 ± 1.1C	25.8 ± 1.3BC	0.44 ± 0.03B	59.6 ± 4.4BC	163.8 ± 3.8B	62.7 ± 0.6A
	E−	32.7 ± 0.9D	23.9 ± 1.6C	0.44 ± 0.03B	54.9 ± 2.3C	143.1 ± 9.3C	62.8 ± 0.7A
200 mM ASS	E+	31.0 ± 0.7DE	25.6 ± 1.8BC	0.33 ± 0.03C	79.7 ± 9.4A	135.0 ± 2.6C	49.5 ± 2.9B
	E−	29.8 ± 0.7E	22.4 ± 1.0C	0.31 ± 0.02C	72.5 ± 3.3AB	131.0 ± 3.6C	46.7 ± 2.6B

E, endophyte; NSS, neutral salt stress; ASS, alkali salt stress. Different uppercase letters following the figures in the same columns indicate significant differences at p < 0.05.

**Figure 2:**
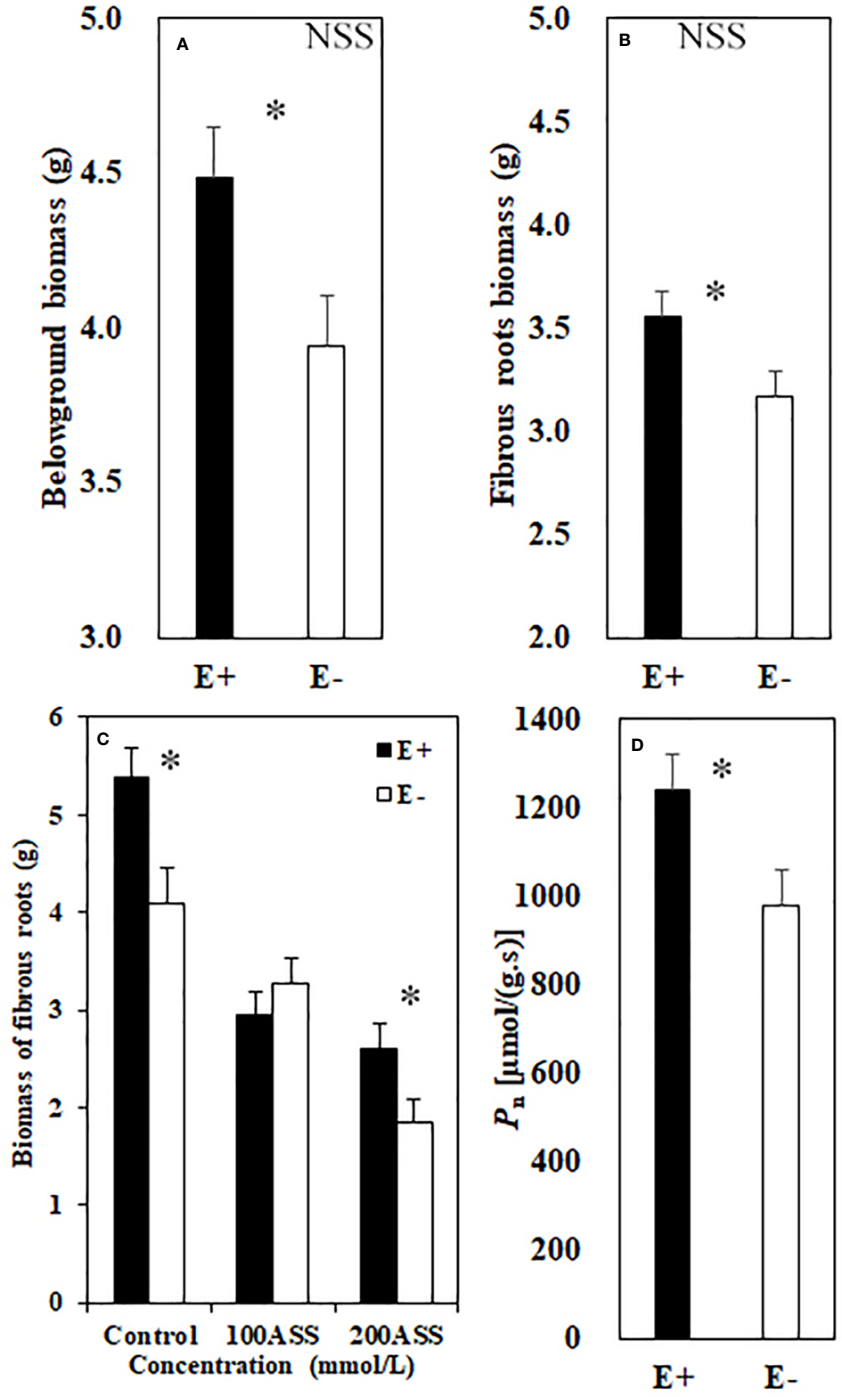
Belowground biomass **(A)** and fibrous root biomass **(B)** under neutral salt stress (NSS) and fibrous root biomass **(C)** under alkali salt stress (ASS) of endophyte infected (E+) and uninfected (E−) *L. chinensis*. *P*_n_ rate **(D)**. Values are mean ± standard error. * indicates significant difference at *p* < 0.05.

Both neutral and alkali salt stresses significantly inhibited total biomass, above- and belowground biomasses of *L. chinensis*, only alkali salt stress was more serious than neutral salt stress (**Tables 2** and **3**). Under neutral salt treatment, both belowground biomass and fibrous root biomass were significantly higher in E+ plants than those in E− plants. For alkali salt treatment, the fibrous root biomass was also higher in E+ plants than that in E− plants under 200 mmol/L treatment (**Figures 2A–C**). *P*_n_ rates in µmol/(m^2^.s) were not significantly influenced by endophyte status (*p*=0.073) (**Table 3**). However, when SLA was considered together *P*_n_ rates in µmol/(g.s) in E+ plants were significantly enhanced than those in E− plants (*p*=0.025) (**Figure 2D**).

**Table 3:** ANOVA results for the effect of endophyte status and salt stress on biomass allocation of *L. chinensis*.

		Total biomass	Aboveground biomass	Belowground biomass	Fibrous root biomass	Rhizome biomass	Dead leaf biomass	Root to Shoot ratio
E	F	0.857	0.782	5.563	4.952	1.428	0.238	4.405
	*p*	0.362	0.383	**0.025**	**0.033**	0.241	0.629	**0.044**
NSS	F	204.282	188.962	28.043	31.837	4.043	42.501	10.722
	*p*	**<0.001**	**<0.001**	**<0.001**	**<0.001**	**0.015**	**<0.001**	**<0.001**
E×NSS	F	3.016	1.343	2.708	3.652	0.763	1.407	0.148
	*p*	**0.044**	0.278	0.062	**0.023**	0.523	0.259	0.930
E	F	0.324	0.447	2.696	3.875	0.092	0.635	2.057
	*p*	0.575	0.510	0.114	0.061	0.764	0.433	0.164
ASS	F	155.610	164.286	27.845	33.387	3.483	72.518	9.903
	*p*	**<0.001**	**<0.001**	**<0.001**	**<0.001**	**0.047**	**<0.001**	**0.001**
E×ASS	F	1.738	0.564	2.848	3.684	0.380	4.637	0.619
	*p*	0.197	0.576	0.078	**0.040**	0.688	**0.020**	0.547

E, endophyte; NSS, neutral salt stress; ASS, alkali salt stress. Significant effects (p ≤ 0.05) are shown in bold.

### Physiological indicators

**Figure 3:**
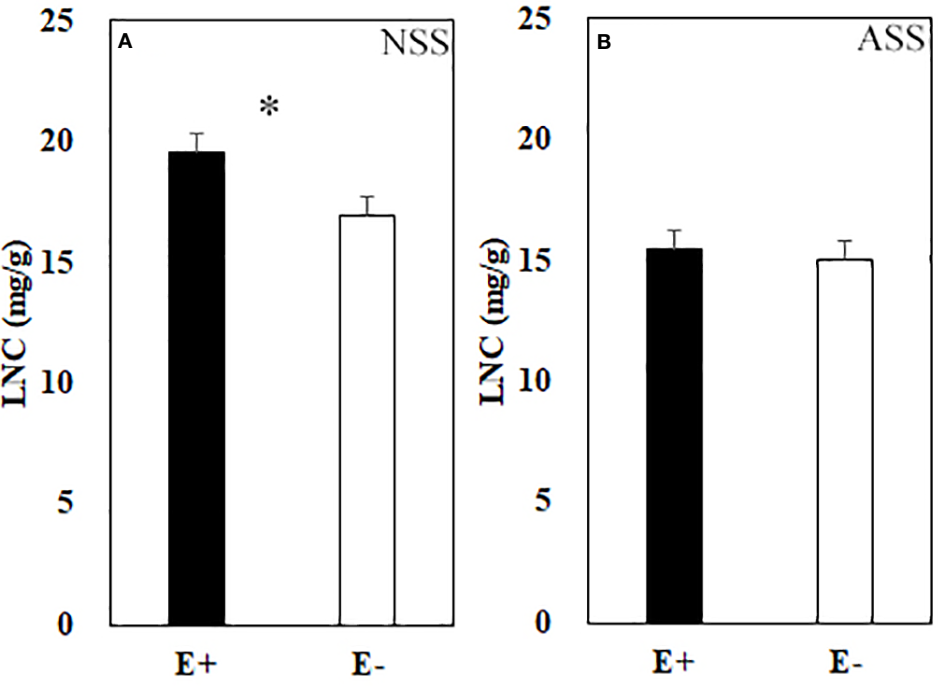
Leaf nitrogen content (LNC) of endophyte infected (E+) and uninfected (E−) *L. chinensis* under neutral salt stress **(A)** and alkali salt stress **(B)**. Values are mean ± standard error. * means significant difference at *p* < 0.05.

Significant effects of neutral salt or alkali salt stresses on chlorophyll *a*, carotenoid and the net photosynthetic rate of leaves were observed in the present study, while these indexes were not influenced by endophyte status (**Table 4**). Endophyte infection significantly enhanced LNC under neutral salt treatment, yet did not affect LNC under alkali salt treatment (**Figures 3A, B**). Both neutral salt and alkaline salt stresses had significant effects on PNUE, while endophyte infection had no significant effect on PNUE.

**Table 4:** ANOVA results for the effect of endophyte status and salt stress on photosynthetic pigment content and photosynthetic indicators.

	Chlorophyll *a*		Chlorophyll *b*		Total Chlorophyll		Chlorophyll *a*/*b*		Carotenoid		*P*_n_		LNC		PNUE	
	F	*p*	F	*p*	F	*p*	F	*p*	F	*p*	F	*p*	F	*p*	F	*p*
E	1.155	0.290	0.034	0.855	0.874	0.357	7.092	**0.012**	1.627	0.211	0.131	0.720	5.540	**0.025**	0.013	0.909
NSS	4.290	**0.012**	1.662	0.195	3.813	**0.019**	5.871	**0.003**	8.145	**<0.001**	70.191	**<0.001**	0.102	0.958	38.37	**<0.001**
E×NSS	0.421	0.739	0.702	0.558	0.466	0.708	1.190	0.329	0.759	0.525	1.587	0.212	5.591	**0.003**	1.466	0.242
E	3.095	0.091	0.665	0.423	2.622	0.118	4.398	**0.047**	3.318	0.081	3.527	0.073	0.128	0.723	1.089	0.307
ASS	6.746	**0.005**	0.880	0.428	5.471	**0.011**	21.95	**0.001**	9.656	**0.001**	94.28	**<0.001**	8.082	**0.002**	47.16	**<0.001**
E×ASS	1.049	0.366	1.009	0.380	1.052	0.365	1.030	0.372	0.923	0.411	0.354	0.705	4.402	**0.024**	0.540	0.590

E, endophyte; NSS, neutral salt stress; ASS, alkali salt stress. Significant effects (p ≤ 0.05) are shown in bold.

### Changes in cation contents and K^+^/Na^+^ ratio

**Figure 4:**
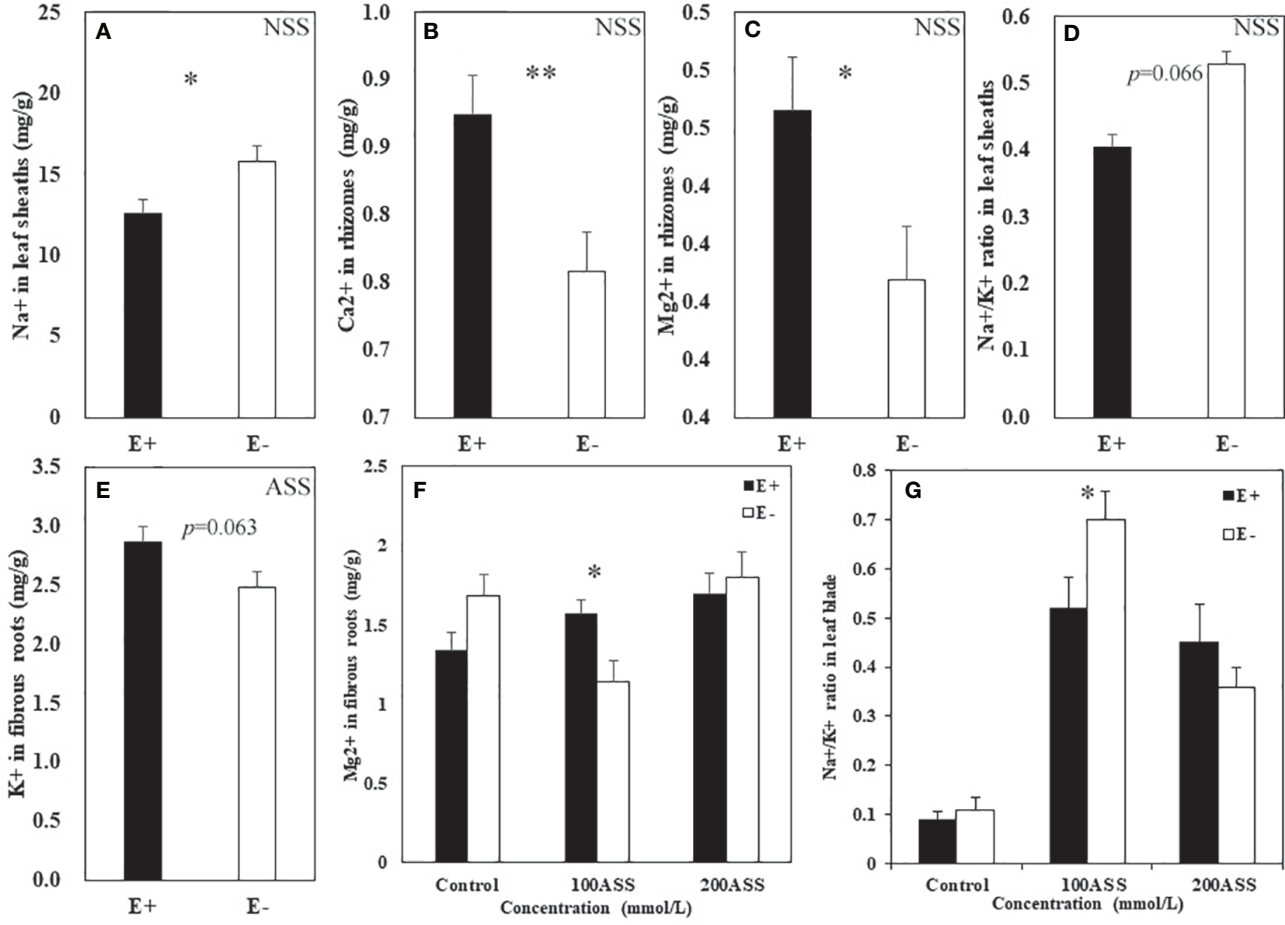
Na^+^, Ca^2+^, Mg^2+^, K^+^ contents and Na^+^/K^+^ ratio in shoots and roots of endophyte infected (E+) and uninfected (E−) *L. chinensis* under neutral salt **(A–D)** and alkali salt **(E–G)** stresses. **(A)** Sheath Na^+^, **(B)** Rhizomes Ca^2+^, **(C)** Rhizomes Mg^2+^, **(D)** Sheath Na+/K+ ratio; **(E)** Fibrous root K^+^, **(F)** Fibrous root Mg^2+^, **(G)** Blade Na+/K+ ratio. Values are mean ± standard error. * and ** indicate significant difference at *p* < 0.05 and *p* < 0.01, respectively.

Both neutral salt stress and alkali salt stresses had significant effects on Na^+^, Ca^2+^, Mg^2+^, K^+^ contents and Na^+^/K^+^ ratio in *L. chinensis* (**Table 5** and **6**). Under neutral salt stress, endophyte infection significantly reduced Na^+^ content in leaf sheaths, while enhanced Ca^2+^ and Mg^2+^contents in rhizomes (**Figures 4A–C**). Endophyte infection tended to decrease Na^+^/K^+^ ratio in the leaf sheath (*p*=0.066) (**Figure 4D**). Under alkali salt stress, endophyte infection tended to enhance K^+^ in the fibrous root. Endophyte infection increased Mg^2+^ content in the fibrous root while decreased Na^+^/K^+^ ratio in the leaf blade in the 100 mmol/L alkali salt treatment (**Figures 4E–G**).

**Table 5:** ANOVA results for the effect of endophyte status and neutral salt stress on ion contents and K^+^/Na^+^ ratio in shoots and roots.

	Leaf blade										Leaf sheath									
	Na^+^		Ca^2+^		Mg^2+^		K^+^		Na^+^/K^+^		Na^+^		Ca^2+^		Mg^2+^		K^+^		Na^+^/K^+^	
	F	*p*	F	*p*	F	*p*	F	*p*	F	*p*	F	*p*	F	*p*	F	*p*	F	*p*	F	*p*
E	<0.001	0.995	0.385	0.539	0.284	0.598	1.795	0.190	0.221	0.641	6.938	**0.013**	1.335	0.257	0.193	0.663	0.278	0.602	3.621	0.066
NSS	24.35	**<0.001**	31.11	**<0.001**	27.79	**<0.001**	1.606	0.207	27.92	**<0.001**	47.24	**<0.001**	33.39	**<0.001**	37.24	**<0.001**	22.03	**<0.001**	34.53	**<0.001**
E×NSS	2.118	0.117	0.620	0.607	1.003	0.404	0.723	0.546	3.085	**0.041**	1.630	0.202	3.216	**0.036**	1.269	0.301	0.464	0.709	1.910	0.148
	Fibrous root										Rhizomes									
		Na^+^	Ca^2+^		Mg^2+^		K^+^		Na^+^/K^+^		Na^+^		Ca^2+^		Mg^2+^		K^+^		Na^+^/K^+^	
	F	*p*	F	*p*	F	*p*	F	*p*	F	*p*	F	*p*	F	*p*	F	*p*	F	*p*	F	*p*
E	1.830	0.186	1.210	0.279	<0.001	0.985	3.713	0.063	0.606	0.442	3.991	**0.054**	8.022	**0.008**	4.965	**0.033**	0.149	0.702	2.378	0.133
NSS	32.74	**<0.001**	2.014	0.132	12.36	**<0.001**	29.38	**<0.001**	62.40	**<0.001**	82.21	**<0.001**	5.459	**0.004**	9.804	**<0.001**	21.34	**<0.001**	27.58	**<0.001**
E×NS	1.660	0.195	0.724	0.545	2.730	0.060	1.531	0.225	1.813	0.165	2.679	0.064	0.337	0.798	3.574	**0.025**	0.528	0.666	0.551	0.651

E, endophyte; NSS, neutral salt stress. Significant effects (p ≤ 0.05) are shown in bold.

**Table 6:** ANOVA results for the effect of endophyte status and alkali salt stress on ion contents and K^+^/Na^+^ ratio in shoots and roots.

	Leaf blade										Leaf sheaths									
	Na^+^		Ca^2+^		Mg^2+^		K^+^		Na^+^/K^+^		Na^+^		Ca^2+^		Mg^2+^		K^+^		Na^+^/K^+^	
	F	*p*	F	*p*	F	*p*	F	*p*	F	*p*	F	*p*	F	*p*	F	*p*	F	*p*	F	*p*
E	0.221	0.643	0.011	0.916	0.496	0.488	2.053	0.165	0.476	0.497	0.022	0.883	4.208	**0.051**	1.672	0.208	0.166	0.687	0.075	0.787
ASS	19.38	**<0.001**	28.66	**<0.001**	17.97	**<0.001**	6.493	**0.006**	49.62	**<0.001**	110.34	**<0.001**	38.05	**<0.001**	23.51	**<0.001**	50.47	**<0.001**	57.09	**<0.001**
E×ASS	0.196	0.823	0.289	0.752	0.849	0.440	1.663	0.211	3.355	**0.052**	3.367	0.051	2.086	0.146	0.624	0.544	0.046	0.955	1.477	0.248
	Fibrous roots										Rhizomes									
		Na^+^	Ca^2+^		Mg^2+^		K^+^		Na^+^/K^+^		Na^+^		Ca^2+^		Mg^2+^		K^+^		Na^+^/K^+^	
	F	*p*	F	*p*	F	*p*	F	*p*	F	*p*	F	*p*	F	*p*	F	*p*	F	*p*	F	*p*
E	0.329	0.571	0.072	0.790	0.253	0.620	3.697	0.063	0.064	0.802	1.296	0.266	0.002	0.961	0.004	0.952	1.995	0.171	0.498	0.487
ASS	102.46	**<0.001**	6.609	**0.005**	3.364	**0.052**	186.4	**<0.001**	87.34	**<0.001**	45.83	**<0.001**	35.39	**<0.001**	38.08	**<0.001**	221.7	**<0.001**	36.00	**<0.001**
E×ASS	0.522	0.600	1.044	0.367	3.039	0.067	7.252	**0.003**	0.054	0.947	1.530	0.237	1.389	0.269	2.317	0.120	1.248	0.305	0.369	0.695

E, endophyte; ASS, alkali salt stress. Significant effects (p ≤ 0.05) are shown in bold.

## Discussion

Both alkali salt and neutral salt stresses involved in deleterious effects of salinity on plant growth; the adverse effects of high pH alkaline solutions on plant growth were more severe than those of low pH salt treatments. High pH is the key characteristic of alkali salt stress that is different from salt stress. The responses of *L. chinensis* were significantly correlated not only with salinity but also with the pH of the alkalinity treatment. It has been reported that alkali salt produced a stronger stress than neutral salt in both halophytes and glycophytes (Yang et al., 2009; Li et al., 2010; Gong et al., 2014). In the present study, *L. chinensis* survived under 400 mmol/L neutral salt treatment, yet died under 300 mmol/L alkali salt treatment, which was consistent with previous reports.

It has been well documented that *Epichloë* endophyes could ameliorate drought stress for both agronomically important forage species (Clay and Schardl, 2002; Saikkonen et al., 2006) and wild grass species (Ren and Clay, 2009; Liu et al., 2017). In the hydroponic experiment, the beneficial effects of *Epichloë* endophytes on NaCl tolerance has been demonstrated in several studies (Reza Sabzalian and Mirlohi, 2010; Yin et al., 2014; Chen et al., 2021). However, expressions of salinity tolerance in hydroponic systems might different with the performance in the soil. Tavakkoli et al. (2010); Tavakkoli et al. (2012) compared salt tolerance of barley under three different growing conditions (hydroponics, potted soil and a naturally saline field) and found that salt tolerance differences between genotypes were expressed when grown in the soil, but cannot be discerned when grown in the hydroponic system. As for plants grown in the potted soil or in the naturally saline field, they expressed similar degrees of salt tolerance. In the present study, we found that endophyte infection significantly increased plant height, leaf length and fibrous root biomass under both neutral and alkali salt stresses, indicating that *Epichloë* endophytes can improve the host resistance to both neutral salt as well as alkali salt grown in the potted soil, only their beneficial effect was more obvious under neutral salt stress than under alkali salt stress.

The mechanisms involved in endophyte-associated salt tolerance of the host have not been documented. According to reported studies and our research, the following possible reasons were proposed. Firstly, endophyte infection could improve root absorption that may alleviate drought and nutrients deficiency during salt stresses. Arbuscular mycorrhizal fungi has been reported to enhance plant growth under salt stress mainly through an extensive hyphal network which allowed enhancement of water and nutrient acquisition (Plenchette and Duponnois, 2005). *Epichloë* endophytes can also increase root absorption (Malinowski et al., 1999; Crush et al., 2004) in response to water stress. In the present study, *L. chinensis* produces two structurally and functionally different organs belowground: fibrous roots that play a central role for root absorption, and rhizomes that serve mainly as translocation conduits and overwinter storage. We found that endophyte infection significantly enhanced fibrous root biomass under both neutral and alkali salt stresses. Although we did not find that endophyte infection improved leaf water content of the host, we did find that endophyte infection increased LNC under neutral salt stress, which was usually negatively influenced by salt stress (Frechilla et al., 2001).

Another mechanism used by *Epichloë* endophytes to promote salt tolerance of the host may be the regulation of plant nutrition. The Na^+^ ion is the main toxic ion in salinized soil. Unlike Na^+^, K^+^ plays a key role in many physiological processes vital to plant growth. Under salt stress, a good balance of Na^+^/K^+^ ratio is important for maintaining ion balance and a number of enzymatic processes, and thus is a potential indicator of salt tolerance in the plants (Munns and Tester, 2008; Abdelhamid et al., 2010; Tomar and Agarwal, 2013). Arbuscular mycorrhizal fungi has been found to play a significant role in sustaining a high K^+^/Na^+^ ratio in plants that were exposed to salt stress (Selvakumar and Thamizhiniyan, 2011; Zhang et al., 2011; Kadian et al., 2013; Fariduddin et al., 2014). In the present study, we found that endophyte infection tended to decrease Na^+^/K^+^ ratio in leaf sheath under neutral salt stress, and also reduced Na^+^/K^+^ ratio in leaf blade in the 100 mmol/L alkali salt treatment.

The accumulations of Ca^2+^ and Mg^2+^ in plants are usually inhibited by salt stress (Khan et al., 1999; Aziz and Khan, 2001). Arbuscular mycorrhizal fungi has been documented to increase the uptake and concentration of Ca^2+^ in different plants (Elhindi et al., 2017; Cui et al., 2019). Rahman and Saiga (2005) reported that endophyte-infected tall fescue had a greater ability to take up Ca^2+^ and Mg^2+^, compared to uninfected plants under normal growth conditions. Bayat et al. (2009) found that endophytes increased Ca^2+^ content in tall fescue under drought stress. In the present study, endophyte infection increased Ca^2+^ and Mg^2+^ contents in the rhizome under neutral salt stress while increased Mg^2+^ content in the fibrous root in 100 mmol/L alkali salt treatment. It is well known that Mg^2+^ is the key component of chlorophyll. The Ca^2+^ ion can maintain membrane stability, help to form cell walls, and take part in signal transduction. Here, increased Mg^2+^ in response to endophyte infection may be related to alleviate chlorophyll breakdown, while increased Ca^2+^ may be related to membrane stability and signal transduction (Cui et al., 2019; Thor, 2019).

## Conclusion

There are over 932 million hectares of land suffering salinization and alkalization around the world (Rengasamy, 2006). It is estimated that soil salinization caused a total loss of US$27.3 billion, with a direct impact on the global economy (Meena et al., 2017). This study demonstrated that endophyte infection could enhance host tolerance to both neutral salt and alkali salt stresses. The significant effect of endophyte infection was decreasing Na^+^/K^+^ ratio, increasing Ca^2+^and Mg^2+^ contents, and thus promoted leaf and fibrous root growth. It is estimated that at least 30% of approximately 3000 pooid grass species harbor systemic endophytes, and most can develop this mutualistic association (Leuchtmann, 1992). In this respect, the potential application of endophytes to mitigate negative salt stress impact would be a more favorable choice. Certainly, this experiment was conducted using soil in pots. Further comparisons of E+ and E− plants under natural field stress conditions will help to verify the potential use of grass-endophyte symbiota in phytoremediation of saline-alkaline soils.

## Data availability statement

The raw data supporting the conclusions of this article will be made available by the authors, without undue reservation.

## Author contributions

LY and MW did experimental work. GW analysed the data and drafted the manuscript. AR designed the experiments and wrote the manuscript. All authors contributed to the article and approved the submitted version.

## Funding

This work was supported by the National Natural Science Foundation of China (31971425).

## Acknowledgments

We greatly appreciate the support of Abaga banner grassland workstation for their invaluable assistance on this experiment.

## Conflict of interest

The authors declare that the research was conducted in the absence of any commercial or financial relationships that could be construed as a potential conflict of interest.

## Publisher’s note

All claims expressed in this article are solely those of the authors and do not necessarily represent those of their affiliated organizations, or those of the publisher, the editors and the reviewers. Any product that may be evaluated in this article, or claim that may be made by its manufacturer, is not guaranteed or endorsed by the publisher.
